# IL-4 Receptor α Chain Protects the Kidney Against Tubule-Interstitial Injury Induced by Albumin Overload

**DOI:** 10.3389/fphys.2020.00172

**Published:** 2020-02-27

**Authors:** Diogo B. Peruchetti, João Luiz Silva-Filho, Rodrigo P. Silva-Aguiar, Douglas E. Teixeira, Christina M. Takiya, Mariana C. Souza, Maria das Graças Henriques, Ana Acacia S. Pinheiro, Celso Caruso-Neves

**Affiliations:** ^1^Instituto de Biofísica Carlos Chagas Filho, Universidade Federal do Rio de Janeiro, Rio de Janeiro, Brazil; ^2^Instituto de Tecnologia em Fármacos, Fundação Oswaldo Cruz, Rio de Janeiro, Brazil; ^3^Rio de Janeiro Innovation Network in Nanosystems for Health – NanoSAUìDE/FAPERJ, Rio de Janeiro, Brazil; ^4^Instituto Nacional de Ciência e Tecnologia em Medicina Regenerativa, INCT-Regenera, Conselho Nacional de Desenvolvimento Científico e Tecnológico/MCTIC, Rio de Janeiro, Brazil

**Keywords:** IL-4, tubule-interstitial injury, albumin overload, renal disease, proteinuria, albumin endocytosis, pro-inflammatory response, acute kidney injury

## Abstract

Increasing evidence has highlighted the role of tubule-interstitial injury (TII) as a vital step in the pathogenesis of acute kidney injury (AKI). Incomplete repair of TII during AKI could lead to the development of chronic kidney disease. Changes in albumin endocytosis in proximal tubule epithelial cells (PTECs) is linked to the development of TII. In this context, interleukin (IL)-4 has been shown to be an important factor in modulating recovery of TII. We have studied the possible role of IL-4 in TII induced by albumin overload. A subclinical AKI model characterized by albumin overload in the proximal tubule was used, without changing glomerular function. Four groups were generated: (1) CONT, wild-type mice treated with saline; (2) BSA, wild-type mice treated with 10 g/kg/day bovine serum albumin (BSA); (3) KO, IL4Rα^–/–^ mice treated with saline; and (4) KO + BSA, IL4Rα^–/–^ mice treated with BSA. As reported previously, mice in the BSA group developed TII without changes in glomerular function. The following parameters were increased in the KO + BSA group compared with the BSA group: (1) tubular injury score; (2) urinary γ-glutamyltransferase; (3) CD4^+^ T cells, dendritic cells, macrophages, and neutrophils are associated with increases in renal IL-6, IL-17, and transforming growth factor β. A decrease in M2-subtype macrophages associated with a decrease in collagen deposition was observed. Using LLC-PK1 cells, a model of PTECs, we observed that (1) these cells express IL-4 receptor α chain associated with activation of the JAK3/STAT6 pathway; (2) IL-4 alone did not change albumin endocytosis but did reverse the inhibitory effect of higher albumin concentration. This effect was abolished by JAK3 inhibitor. A further increase in urinary protein and creatinine levels was observed in the KO + BSA group compared with the BSA group, but not compared with the CONT group. These observations indicate that IL-4 has a protective role in the development of TII induced by albumin overload that is correlated with modulation of the pro-inflammatory response. We propose that megalin-mediated albumin endocytosis in PTECs could work as a sensor, transducer, and target during the genesis of TII.

## Introduction

There is a strict correlation between AKI and CKD, a public health problem with a high level of morbidity and mortality (Liyanage et al., 2015; Negi et al., 2018). Increasing evidence has highlighted the role of TII as a vital step in the pathogenesis of AKI (Nakhoul and Batuman, 2011; Venkatachalam et al., 2015; Chevalier, 2016; Strausser et al., 2018). The incomplete repair of tubular injury during the course of AKI could lead to the development of CKD (Venkatachalam et al., 2015; Strausser et al., 2018). Several studies have demonstrated an important role of albumin overload in the PT in the development of TII (Gorriz and Martinez-Castelao, 2012; Erkan, 2013; Portella et al., 2013; Abreu et al., 2014; Landgraf et al., 2014; Teixeira et al., 2019). This mechanism requires changes in the albumin endocytosis machinery (Nakhoul and Batuman, 2011; Gorriz and Martinez-Castelao, 2012; Landgraf et al., 2014; Teixeira et al., 2019).

It is well known that albumin reabsorption in PT epithelial cells (PTECs) occurs by receptor-mediated endocytosis with megalin as the main receptor involved in this process (Gorriz and Martinez-Castelao, 2012; Erkan, 2013; Eshbach and Weisz, 2017). Some studies have shown that megalin works as sensor and integrator between changes in albumin concentration and PTEC injury (Caruso-Neves et al., 2006; Erkan, 2013; Peruchetti et al., 2014; Nielsen et al., 2016). This process is mediated by different cellular mechanisms triggered by changes in megalin expression (Caruso-Neves et al., 2006; Peruchetti et al., 2014). In a previous work, our group showed that megalin knockdown in LLC-PK1 cells mimicked the effect of pathologic albumin concentration on different signaling pathways, which could be correlated with cell apoptosis and a pro-inflammatory response (Caruso-Neves et al., 2006; Peruchetti et al., 2014). In addition, changes in megalin expression have been correlated with TII in Fanconi syndrome as well as autoimmune renal diseases (Piwon et al., 2000; Christensen et al., 2003; Batuman, 2007; Nakhoul and Batuman, 2011; Vicinanza et al., 2011; Gaide Chevronnay et al., 2014; Eshbach and Weisz, 2017; Wang et al., 2017; Cez et al., 2018).

The complete repair of tubular injury depends on the complex network involved in the early and resolution phase of the inflammatory response (Huen and Cantley, 2017; Lee et al., 2017). In this context, CD4+ T cells and macrophages have been shown to be important players in the initial and resolution phase of the inflammatory response during the development of TII (Huen and Cantley, 2017; Lee et al., 2017). Interestingly, it was shown that PTECs also participate in the activation of a pro-inflammatory response, secreting cytokines and chemokines after a specific signal such as exposure to albumin overload (Zoja et al., 1998; Donadelli et al., 2003; Takaya et al., 2003; Tang et al., 2003; Reich et al., 2005; Pearson et al., 2008; Gorriz and Martinez-Castelao, 2012; Erkan, 2013; Landgraf et al., 2014; Teixeira et al., 2019). PTECs could work as APCs, inducing an inflammatory phenotype with increasing CD4+ T cell responses (Breda et al., 2019).

Interleukin (IL)-4 has been shown to be an important factor in modulating the recovery of tubular injury in renal disease (Cao et al., 2011; Yan et al., 2015; Liang et al., 2017; Zhang et al., 2017). Using an AKI animal model, Zhang et al. (2017) showed that IL-4 induces polarization of the M2 macrophage phenotype, which was correlated with the recovery of tubular injury. In agreement, it was shown that STAT6-deficient mice develop more severe injury in an ischemia-reperfusion AKI animal model (Yokota et al., 2003). On the other hand, using a unilateral ureteral obstruction model, Liang et al. (2017) showed that the IL-4 receptor α chain/STAT6 pathway activates bone marrow-derived fibroblasts, leading to the development of fibrosis and aggravation of renal disease. These apparent contradictory results indicate that the final effect of IL-4 is more complex than has been described.

Based on this scenario, we decided to study the effect of IL-4 receptor α (IL-4Rα) chain on TII induced by albumin overload. To address this issue, a subclinical AKI (AKIsub) animal model in BALB/c (wild-type) or IL-4Rα chain deficient (IL4Rα^–/–^) mice was used. In addition, LLC-PK1 cells were used as a model of PTECs to study the cellular response. It was observed that the IL-4Rα chain protects the development of TII induced by albumin overload. This process involves modulation of the albumin endocytosis machinery and a pro-inflammatory response.

## Materials and Methods

### Reagents

Bovine serum albumin (BSA) fraction V (delipidated - #A9647), BSA conjugated to fluorescein isothiocyanate (BSA-FITC), CP-690550-10, collagenase and protease inhibitor cocktail (no. I3786) were purchased from Sigma-Aldrich (St. Louis, MO, United States). MK-2206 was purchased from Selleckchem (Houston, TX, United States). Sensiprot kit (ref. 36) was purchased from Labtest (Lagoa Santa, MG, Brazil). Creatinine kit (ref. 335) was purchased from Gold Analisa (Belo Horizonte, MG, Brazil). γ-GT kit (ref. K080) was purchased from Bioclin (Belo Horizonte, MG, Brazil). Recombinant porcine IL-4 was purchased from R&D Systems (Minneapolis, MN, United States). Polyclonal phospho-STAT6 (Tyr-641), polyclonal STAT6, polyclonal phospho-STAT3 (Ser-727), polyclonal STAT3, polyclonal phospho-ERK1/2 (Thr-202/Tyr-204), polyclonal phospho-PKB (Ser-473), polyclonal PKB, polyclonal ERK1/2, polyclonal inducible nitric oxide synthase (iNOS), polyclonal β-actin, and HRP-conjugated anti-rabbit IgG antibodies were purchased from Cell Signaling Technology (Danvers, MA, United States). Polyclonal Lrp2/megalin and polyclonal nephrin antibodies were purchased from Abcam (Cambridge, MA, United States). Polyclonal IL-4Rα and podocin antibodies were purchased from Santa Cruz Biotechnology (Dallas, TX, United States). Monoclonal mouse IgG1 anti-mouse arginase-1 (clone 19, BD Transduction Laboratories), monoclonal rat IgG2a anti-mouse IA-IE (clone 2G9; Pharmingen), PE-conjugated rat IgG2b anti-mouse Ly-6G and Ly-6C (clone RB6-8C5; Pharmingen), PE-conjugated rat IgG2b anti-mouse CD11b (clone M1/70; Pharmingen), and PE-conjugated hamster IgG1 anti-mouse CD11c (clone HL3; Pharminge) were purchased from BD Bioscience (São Paulo, Brazil). Biotinylated anti-rabbit or anti-mouse IgG was purchased from Agilent Technologies (Santa Clara, CA, United States). Dulbecco’s modified Eagle’s medium (DMEM), phosphate-buffered saline (PBS), fetal bovine serum (FBS), 3,30-diaminobenzidine, UltraPure N, N’-methylene bisacrylamide, FITC-conjugated rat IgG2b anti-mouse CD4 (clone GK1.5; eBioscience), and PerCP-Cyanine5.5-conjugated IgG2a anti-mouse CD8a (clone 53-6.7; eBioscience) were purchased from Thermo Fisher Scientific (Waltham, MA, United States). LLC-PK1 cells were purchased from the ATCC (Rockville, MD, United States). All other reagents were of the highest purity available.

### Animals

Male BALB/c mice (weighing 20–25 g), 8–11 weeks old, were used in all experiments. Wild-type (WT) BALB/c mice were obtained from the Institute of Science and Technology in Biomodels (ICTB) of the Oswaldo Cruz Foundation (FIOCRUZ), Rio de Janeiro, Brazil. IL-4Rα chain deficient mice (IL4Rα-/-, BALB/c-Il4ratm1Sz/J; backcrossed to BALB/c at least seven generations) were obtained from The Jackson Laboratories. All mice were housed, bred, and maintained in the animal care facility at the Federal University of Rio de Janeiro. The animals were accommodated in an air-conditioned environment (22–24°C) in a regular 12-h light/dark cycle with water and standard chow *ad libitum*. The presence or absence of any adverse clinical signs associated to this mouse strain was monitored as described previously (Teixeira et al., 2019). All procedures involving the handling of animals were conducted in accordance with the National Institutes of Health (NIH) Guide for the Care and Use of Laboratory Animals and were approved by the Institutional Ethics Committee of the Federal University of Rio de Janeiro (protocol number IBCCF098).

### Subclinical AKI Animal Model

The AKIsub animal model was developed as described previously (Portella et al., 2013; Abreu et al., 2014; Landgraf et al., 2014; Peruchetti et al., 2019; Teixeira et al., 2019). Briefly, WT or IL4Rα-/- mice were used to generate four experimental groups: (1) CONT, WT mice treated with saline (used as vehicle); (2) BSA, WT mice treated with 10 g/kg/day BSA via intraperitoneal injection (ip) for 7 consecutive days; (3) KO, IL4Rα-/- mice treated with saline; and (4) KO + BSA, IL4Rα-/- mice treated with BSA. The animals were kept in the metabolic cage during all experiment and 24-h urine was collected at day 7. At the end of day 7, the animals were euthanized using a mixture of ketamine (240 mg/kg) and xylazine (15 mg/kg). The kidneys were removed and used for: (1) histomorphometry; (2) detection of specific proteins through immunohistochemistry and immunoblotting; (3) level of cytokines; and (4) immune cell infiltration.

### Cell Culture

LLC-PK1 cells, a porcine PTEC line, were grown at 37°C in 5% CO2 in low-glucose DMEM supplemented with 10% FBS and 1% penicillin/streptomycin (Caruso-Neves et al., 2005, 2006; Lara et al., 2010; Lindoso et al., 2011; Peruchetti et al., 2011, 2014, 2018, 2019; Landgraf et al., 2014; Arnaud-Batista et al., 2016; Teixeira et al., 2019). After reach the confluence (85–90%), the serum-starved cells were treated with different compounds as indicated in the figure legends. Then, it was determined the rate of albumin endocytosis and detection of specific proteins through immunoblotting.

### Renal Function Analysis

The renal function parameters were obtained as previously published (Portella et al., 2013; Abreu et al., 2014; Landgraf et al., 2014; Silva-Filho et al., 2016; Silva-Aguiar et al., 2018; Teixeira et al., 2019). Urine samples were used to assess the levels of γ-glutamyltransferase (γ-GT) activity, proteins and creatinine using commercial kits, according to the manufacturer’s instructions. The creatinine clearance (CCr) and the ratio of urinary protein to creatinine (UPCr) were calculated using those parameters.

### Histologic and Immunohistochemistry Studies

The histology and immunohistochemistry studies were performed as previously (Portella et al., 2013; Abreu et al., 2014; Landgraf et al., 2014; Silva-Filho et al., 2016; Silva-Aguiar et al., 2018; Teixeira et al., 2019). 5-μm-thick kidney sections stained with periodic acid-Schiff (PAS) were used for analysis of both glomerular and tubular structures. These sections were also used to determine megalin expression through immunohistochemistry. 8-μm-thick kidney sections were used to determine the cortical collagen deposition after Picrosirius red staining.

30 images were acquired randomly using a Nikon 80i eclipse microscope (Nikon, Japan) followed by quantification analysis using Image-Pro Plus software (Media Cybernetics, Rockville, MD, United States). To evaluate the possible changes in glomerular structure, both glomerular cellularity (number of glomerular cells) and mesangial expansion (intensity of mesangial matrix at glomerular tuft) were determined. To assess possible changes in tubular structure, a tubular injury score was used (Grgic et al., 2012). The red fibers intensity was measured in PicroSirius red-stained slices to determine the levels of collagen deposition (expressed as percentage of the interstitial area). The intensity of megalin-positive staining (expressed as arbitrary units) was measured as a ratio between the intensity of the positive area for megalin and total tissue area. All quantifications were performed in a blinded manner.

### Immunoblotting

Immunoblotting was performed as described previously (Caruso-Neves et al., 2006; Lara et al., 2008; Peruchetti et al., 2011, 2014, 2018; Landgraf et al., 2014; Arnaud-Batista et al., 2016; Silva-Filho et al., 2016; Ribeiro et al., 2018; Silva-Aguiar et al., 2018; Teixeira et al., 2019). Briefly, protein samples were obtained from LLC-PK1 cell lysate or renal cortex homogenate. Cell lysate and homogenate were clarified by centrifugation at 15,000 × *g* for 10 min at 4°C in an ice-cold solution described previously in the works above. The Folin phenol method was used to assess the total protein concentration (Lowry et al., 1951).

Proteins (30–60 μg) were resolved on 5% or 9% SDS-PAGE and transferred to PVDF membranes (Millipore). After blockade of non-specific binding, the membranes were incubated with specific primary antibodies and their respective HRP-conjugated IgG secondary antibodies, according to the manufacturer’s instructions. ECL Prime was used to detect the proteins of interest (Peruchetti et al., 2018; Ribeiro et al., 2018; Silva-Aguiar et al., 2018; Teixeira et al., 2019). All the images were obtained using the Image Quant LAS4000 Image processing system (GE Healthcare Life Sciences, Pittsburgh, PA, United States) and processed by adjusting the brightness and contrast using NIH ImageJ software (version 1.6.0). It is important to mention that this image processing method was applied to every pixel in the original image without changing the information illustrated.

#### Assessment of Albumin Endocytosis

The rate of albumin endocytosis was assessed through the measurement of BSA-FITC uptake as described previously (Caruso-Neves et al., 2005; Peruchetti et al., 2018; Silva-Aguiar et al., 2018; Teixeira et al., 2019). Briefly, the cells were incubated with Ringer solution (12.4 mM HEPES–Tris (pH 7.4), 140 mM NaCl, 2.7 mM KCl, 1.8 mM CaCl2, 1 mM MgCl2, 5 mM glucose) containing 30 μg/mL BSA-FITC at 37°C for 30 min. After the reaction, the unbound BSA-FITC was washed extensively with ice-cold Ringer solution. Then, the cells were lysed using 0.1% Triton X-100 and the cell-associated fluorescence was determined using a microplate spectrofluorimeter (SpectraMax M2; Molecular Devices, Sunnyvale, CA, United States). BSA-FITC-specific uptake was calculated as the difference between the fluorescence intensity in the absence and in the presence of 30 mg/mL unlabeled BSA. The data obtained were further normalized by the total protein concentration and, then, expressed as arbitrary units.

#### Assessment of Renal Albumin-FITC Uptake

The renal cortex albumin-FITC uptake was measured as published previously (Silva-Aguiar et al., 2018; Teixeira et al., 2019). Briefly, mice received single intravenous injection of 5 μg/g BSA-FITC. After 15 min, renal cortexes slides were homogenized in ice-cold Ringer solution (20 mM HEPES–Tris (pH 7.4), 5 mM D(+)-glucose, 2.7 mM KCl, 140 mM NaCl, 1 mM MgCl2, 1.8 mM CaCl2, 1 mM PMSF and protease inhibitor cocktail 1×). After clarification, samples were used to determine the intensity of organ-associated fluorescence (excitation = 480 nm, emission = 520 nm) using SpectraMax M2 (Molecular Devices, Sunnyvale, CA, United States). The specific albumin-FITC uptake was further normalized by total protein concentration. Data were expressed as percentage of the controls.

### Renal Cytokines

The levels of cortical cytokines were evaluated as described (Portella et al., 2013; Landgraf et al., 2014). The concentrations of IFN-γ, IL-4, IL-6, IL-10, IL-17, RANTES, TGF-β, and TNF-α were determined by cytometric bead array (BD Biosciences, San Jose, CA, United States). Data are represented by ratio between ng of cytokine/chemokine and mg of total proteins (ng/mg).

### Immune Cell Infiltration

Immune cell infiltration in the renal tissue was assessed as described previously (Portella et al., 2013). Briefly, kidney thick sections were added in DMEM supplemented with 10 mM HEPES, 10 mM non-essential amino acids, 2 mM L-glutamine, 1 mM sodium pyruvate, 0.05 mM 2-mercaptoethanol, 100 U/mL penicillin, and 100 U/mL streptomycin. Then, the kidney slices were digested in a solution containing 0.1% collagenase (37°C for 60 min) with gentle shaking. After filtration, removal of erythrocytes and clarification, the cells were resuspended and washed with cold PBS. Then, the cells were incubated with Fc block (affinity purified anti-mouse CD16/CD32; eBioscience, San Diego, CA, United States) and with specific antibodies. The acquisition was obtained in FACSCalibur flow cytometer (Becton Dickinson, San Jose, CA, United States) followed by the analysis of surface markers in Summitt software as shown previously (Silva-Filho et al., 2013, 2017; Ribeiro et al., 2018). All data were collected and displayed on a log scale of increasing fluorescence intensity and presented as dot plots. The percentages of T cells (CD4+ and CD8+), macrophages (CD11b+), dendritic cells (CD11c+ IA-IE+) and neutrophils (Ly6G+) were determined in a specific gate.

### Statistical Analysis

All data are expressed as means ± standard deviation (SD). For statistical analysis, it was used the GraphPad Prism 7 (version 7.00, GraphPad Software, San Diego, CA, United States)^1^. For comparison between two experimental groups (as shown in some experiments using LLC-PK1 cells), it was used the unpaired *t*-tests. For three or more experimental groups (as shown in some experiments using LLC-PK1 cells and the AKIsub animal model), it was used the one-way analysis of variance (ANOVA) test followed by the Newman-Keuls post test. *P* < 0.05 is used to determine statistical significance.

## Results

### Tubule-Interstitial Injury Induced by Proximal Tubule Albumin Overload Is Worsened by IL-4Rα Deficiency

**FIGURE 1:**
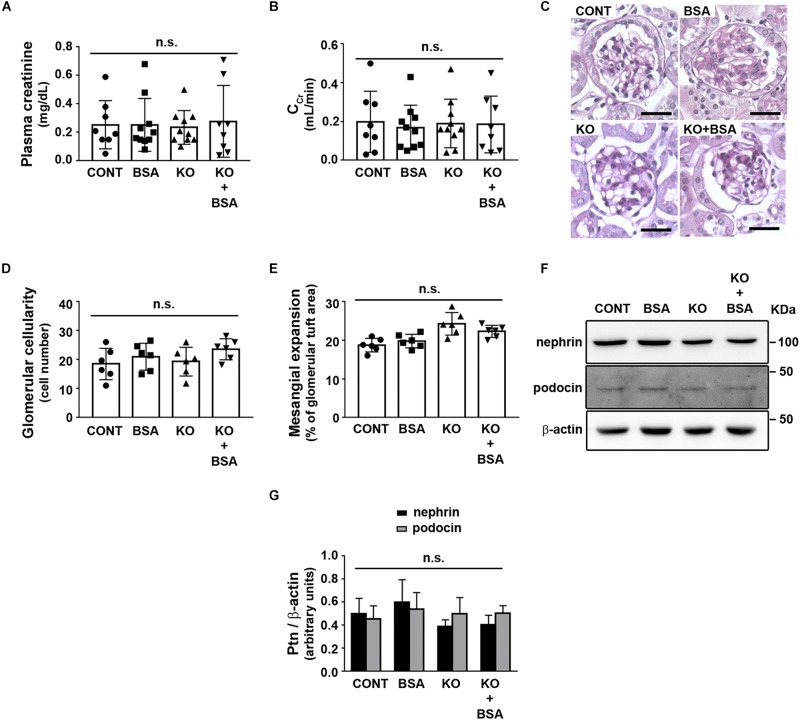
IL-4Rα deficiency with or without subclinical AKI does not change glomerular function. Male BALB/c (wild-type) or IL-4Rα deficient (IL-4Rα^–/–^) mice were used to develop a subclinical AKI animal model through the intraperitoneal injection of 10 g/kg/day BSA over 7 consecutive days (for details see section “Materials and Methods”). Four experimental groups were generated: (1) CONT, wild-type (WT) mice treated with saline (used as vehicle); (2) BSA, WT mice treated with BSA; (3) KO, IL4Rα^–/–^ mice treated with saline; and (4) KO + BSA, IL4Rα^–/–^ mice treated with BSA. **(A,B)** Assessment of glomerular function. **(A)** Plasma creatinine (*n* = 8). **(B)** Creatinine clearance (CCr, *n* = 8). **(C–E)** Evaluation of glomerular structure. **(C)** Representative images of glomeruli (scale bar represents 100 μm). **(D)** Glomerular cellularity (*n* = 6). **(E)** Measurement of mesangial expansion (*n* = 6). **(F,G)** Nephrin and podocin expression. **(F)** Representative images of four independent experiments. β-Actin was used as the load control. Ptn, protein. **(G)** Densitometry analyzes related to **(F)** (*n* = 4). The OD related to nephrin or podocin bands was normalized to the OD related to the β-actin band. The results are expressed as means ±SD. n.s., not significant.

To address the role of IL-4R in the development of TII, we used BALB/c (WT) or IL4Rα^–/–^ mice to generate four different experimental groups as described earlier. Initially, possible change in glomerular function was assessed (Figure 1). It was observed that the plasma creatinine level and creatinine clearance, markers of the glomerular flow rate, were not changed in any experimental groups (Figures 1A,B). In agreement, glomerular cellularity and mesangial expansion as well as podocin and nephrin expression were also not changed (Figures 1C–G).

**FIGURE 2:**
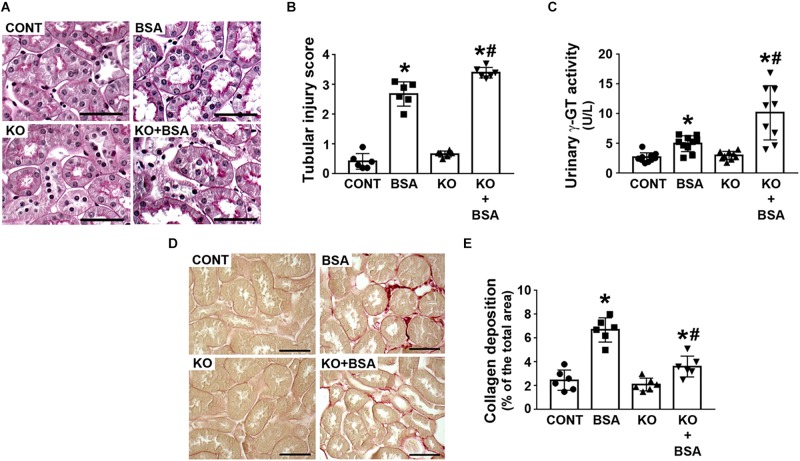
IL-4Rα deficiency worsens tubule-interstitial injury in subclinical AKI. Mice were treated as described in Figure 1. **(A)** Representative images of the cortical tubular region. Kidney slices were stained with periodic acid-Schiff (PAS) (scale bar represents 50 μm). **(B)** Tubular injury score (*n* = 6). **(C)** Urinary γ-GT activity (*n* = 9). **(D)** Representative images of collagen fiber deposits (red color) in the renal cortex. Kidney slices were stained with Sirius red (scale bar represents 50 μm). **(E)** Quantitative analysis related to **(D)** (*n* = 6). The results are expressed as means ±SD. *P < 0.05 versus the CONT group; #*P* < 0.05 versus the BSA group.

The score index for tubular injury in the BSA-treated groups was increased and this effect was more pronounced in the KO + BSA group compared with the BSA group (Figures 2A,B). In agreement, γ-GT activity, a specific marker for PT cell injury, was higher in the KO + BSA group than in the BSA group (Figure 2C). No significant change in the score index for tubular injury or γ-GT activity, marker of PT injury, was observed in the KO group compared with the CONT group. Interestingly, collagen deposition was increased in the BSA-treated groups (BSA and KO + BSA). However, contrary to the results for other parameters, collagen deposition in the KO + BSA group was lower than that in the BSA group (Figures 2D,E).

### Protein Handling in IL-4Rα Deficiency

**FIGURE 3:**
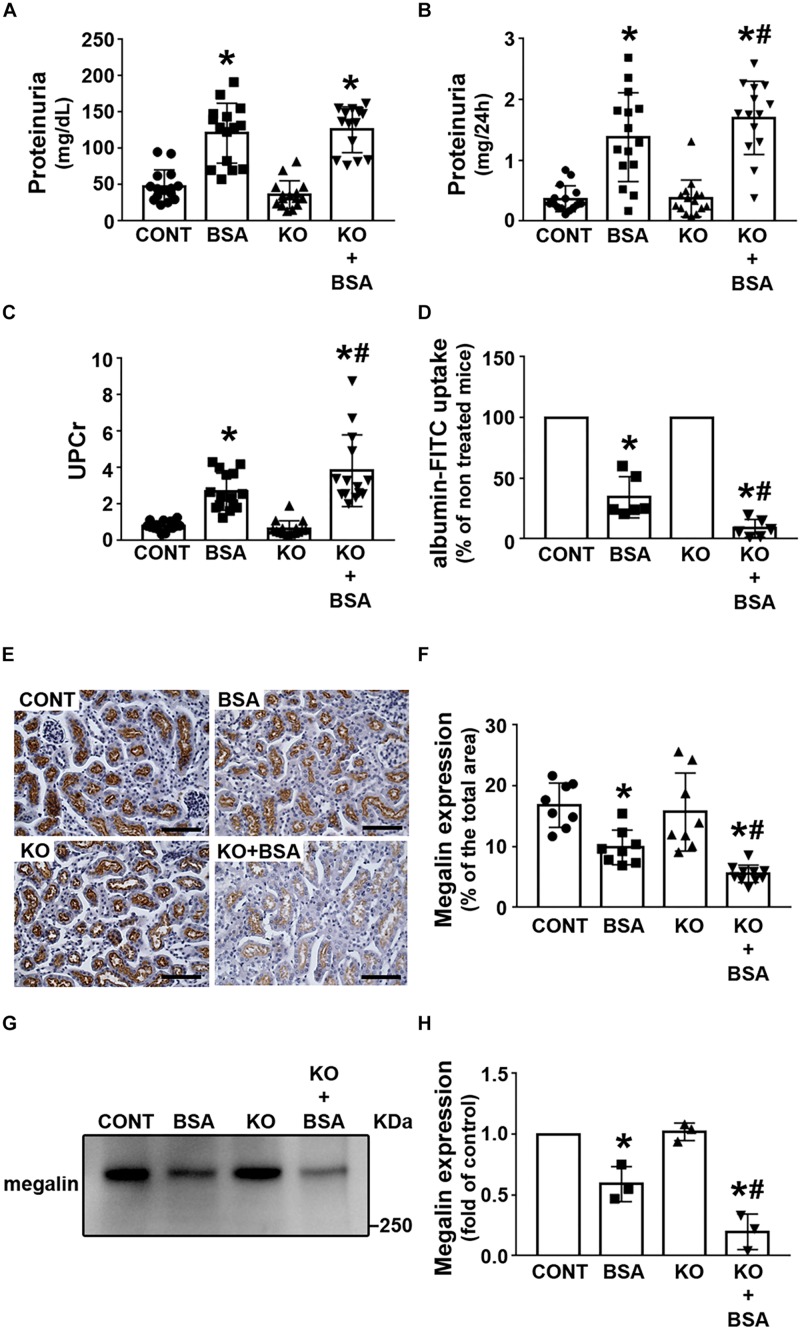
IL-4Rα deficiency worsens proteinuria due to a decrease in megalin expression in subclinical AKI. Mice were treated as described in Figure 1. Measurement of proteinuria [**(A,B)**, *n* = 15] and the ratio of urinary proteins to creatinine [**(C)**, *n* = 15]. **(D)** *In vivo* albumin-FITC uptake (*n* = 6). **(E)** Representative megalin staining in the cortical area (bars represent 100 μm). **(F)** Quantitative analysis related to **(E)**. **(G)** Megalin expression in renal cortex was determined by immunoblotting (*n* = 3). **(H)** Quantitative analysis related to **(G)**. The results are expressed as means ±SD. *P < 0.05 versus the CONT group; #*P* < 0.05 versus the BSA group.

Some groups, including our group, have proposed that there is a correlation among changes in albumin concentration in PT, megalin expression, and TII development (Caruso-Neves et al., 2006; Gorriz and Martinez-Castelao, 2012; Erkan, 2013; Peruchetti et al., 2014; Teixeira et al., 2019). Here, it was observed that proteinuria was higher in the BSA and KO + BSA groups than in the CONT group, but was not changed in the KO group (Figures 3A,B). UPCr was measured to rule out the possible influence of urinary flow on proteinuria (Figure 3C). UPCr was increased in the BSA and KO + BSA groups compared with the CONT group. However, it was significantly higher in the KO + BSA group than in the BSA group, similarly to the trends observed for the score index for tubular injury and γ-GT activity. In the next step, albumin endocytosis in renal cortex was accessed (Figure 3D). It was decreased in BSA and KO + BSA groups when compared with CONT group (Figure 3D). Similarly, the expression of megalin, a protein endocytosis receptor, was also decreased in the BSA and KO + BSA groups (Figures 3E–H). Interestingly, the decrease in albumin endocytosis and megalin expression were more pronounced in the KO + BSA than in BSA group. Together these results indicate that IL-4/IL-4Rα pathway attenuate changes in PT albumin endocytosis machinery in TII induced by PT albumin overload.

### IL-4 Modulates Albumin Endocytosis in Proximal Tubule Cells

**FIGURE 4:**
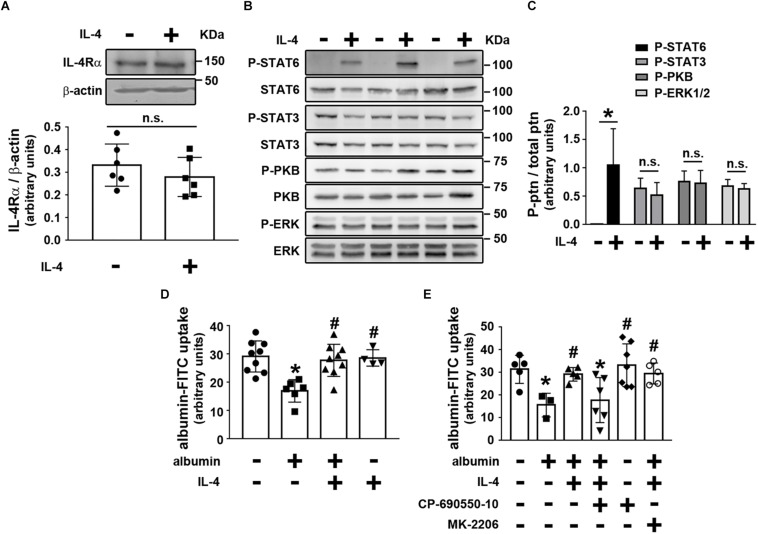
IL-4 avoids the inhibitory effect of higher albumin concentration on albumin endocytosis through activation of the JAK3/STAT6 pathway. LLC-PK1 cells were grown in 24-well plates until 95% confluence was reached. Then, the cells were incubated with different compounds as indicated. **(A)** The effect of 20 ng/mL IL-4 on IL-4Rα chain expression (*n* = 6). β-actin was used as the load control. In upper panel is showed the representative images. In bottom panel is showed the densitometry analyzes. **(B,C)** The effect of IL-4 on the phosphorylation of STAT6 (Tyr641), STAT3 (Tyr705), PKB (Ser473), and ERK (Thr202/Tyr204). **(B)** Representative images of three independent experiments. **(C)** Densitometry analysis related to **(B)** (*n* = 3). Ptn, protein. All phosphorylated fractions of specific proteins were normalized by the total fraction of the respective proteins. **(D)** The effect of IL-4 on the inhibitory effect of 20 mg/mL albumin on albumin endocytosis (*n* = 9). Albumin endocytosis was measured by cell-associated fluorescence using albumin-FITC as a tracer. **(E)** The effect of 10^–7^ M CP-690550-10 (JAK3 inhibitor) or 10^–7^ M MK-2206 (PKB inhibitor) on the effect of IL-4 on albumin endocytosis (*n* = 6). The results are expressed as means ±SD. *P < 0.05 versus control; #*P* < 0.05 versus albumin; n.s., not significant.

We then investigated the possible effect of IL-4 on PT albumin endocytosis. LLC-PK1 cells, a model of PT cells, were used to address this question. Initially, it was shown that LLC-PK1 cells express the IL-4Rα chain, which was not changed when the cells were incubated overnight with 20 ng/mL IL-4 (Figure 4A). Furthermore, overnight incubation of LLC-PK1 cells with IL-4 led to increased STAT6 phosphorylation, associated with the IL-4Rα chain (Figures 4B,C). However, there was no change in the phosphorylation of PKB, STAT3, and ERK1/2.

Next, we wanted to determine if the cellular response triggered by IL-4 in LLC-PK1 cells modulated albumin endocytosis. Initially, incubation of LLC-PK1 cells with 20 ng/mL IL-4 overnight did not change albumin endocytosis (Figure 4D). When the cells were incubated overnight with a higher albumin concentration (20 mg/mL), mimicking the effect of albumin overload in PTs, albumin endocytosis was inhibited in 38% (Figure 4D). Interestingly, this effect was completely reversed by co-incubation of the LLC-PK1 cells with 20 ng/mL IL-4. These results are in accordance with the observation that proteinuria and UPCr were not changed in the KO group, but they were increased in the KO + BSA group. Furthermore, the molecular mechanism underlying the effect of IL-4 on albumin endocytosis involves STAT6 activation since 10^–7^ M CP-690550-10, a JAK3 inhibitor, completely abolished it. On the other hand, MK-2206, a PKB inhibitor, did not change the IL-4 effect (Figure 4E). So far, the results indicate that KO mice further increase TII induced by albumin overload, which could be associated with a decrease in the albumin endocytosis machinery.

### Deficiency of IL-4Rα Enhances the Pro-inflammatory Response During Tubule-Interstitial Injury Induced by Albumin Overload

**FIGURE 5:**
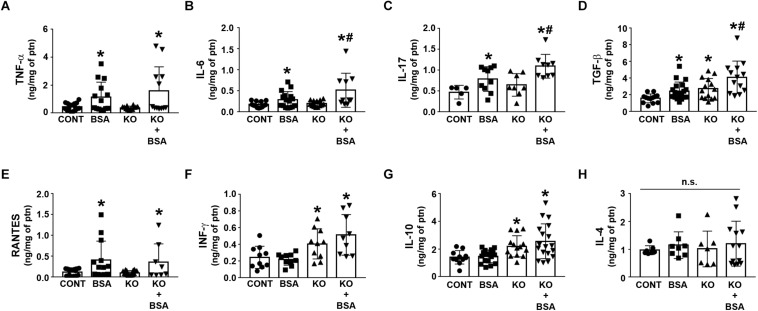
IL-4Rα deficiency enhances renal pro-inflammatory cytokines in subclinical AKI. Mice were treated as described in Figure 1. Measurement of cortical TNF-α [**(A)**, *n* = 12], IL-6 [**(B)**, *n* = 10], IL-17 [**(C)**, *n* = 9], TGF-β [**(D)**, *n* = 13], RANTES [**(E)**, *n* = 8], IFN-γ [**(F)**, *n* = 9], IL-10 [**(G)**, *n* = 18], and IL-4 levels [**(H)**, *n* = 13] were determined by ELISA. The cytokine levels were normalized by the amount of total protein in the same samples. TNF-α, tumor necrosis factor alpha; IL, interleukin; TGF-β, transforming growth factor β; RANTES, regulated on activation, normal T cell expressed and secreted; INF-γ, interferon-γ. The results are expressed as means ±SD. *P < 0.05 versus the CONT group; #*P* < 0.05 versus the BSA group.

Figure 5 shows the cytokines and chemokines in the renal cortex homogenate for different experimental groups. The level of TNF-α, IL-6, IL-17, TGF-β, and RANTES was increased in the BSA-treated groups (BSA and KO + BSA; Figures 5A–E). A further increase in the IL-6, IL-17, TGF-β levels was observed in the KO + BSA group compared with the BSA group. In addition, the level of TGF-β was also increased in the KO group compared with the control group (Figure 5D). The levels of INF-γ and IL-10 were only increased in the KO groups (KO and KO + BSA; Figures 5F,G). On the other hand, the level of IL-4 was not changed in any experimental group (Figure 5H).

**FIGURE 6:**
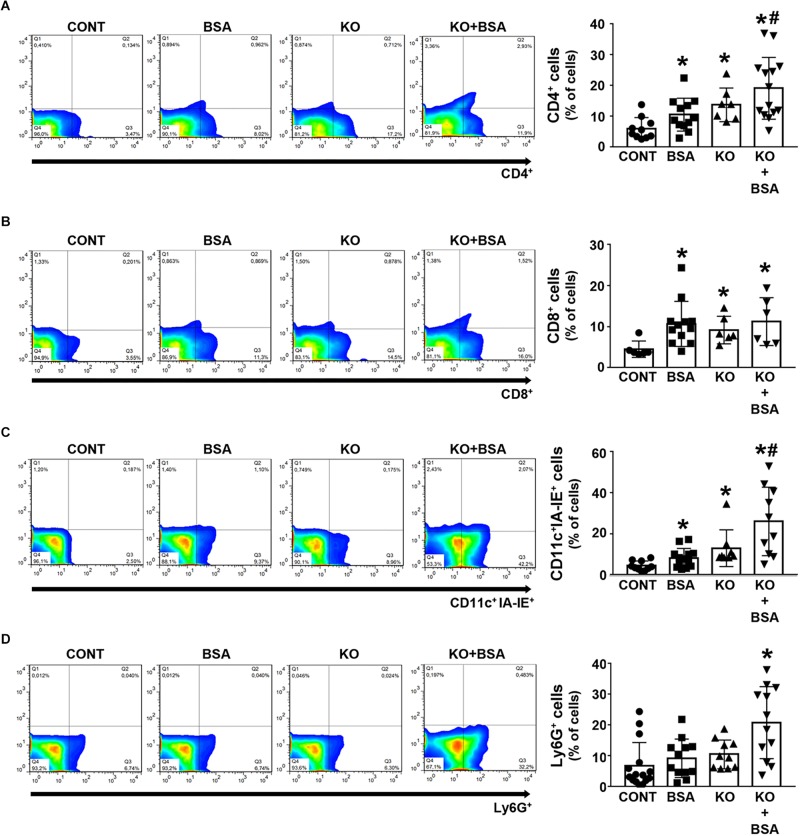
IL-4Rα deficiency potentiates immune cell infiltration in subclinical AKI. Mice were treated as described in Figure 1. The kidneys were perfused with saline and digested with collagenase. The percentage of CD4 + T cells [**(A)**, *n* = 14], CD8 + T cells [**(B)**, *n* = 8], CD11c + IA-IE + dendritic cells [**(C)**, *n* = 10], and Ly6G + neutrophils [**(D)**, *n* = 12] were determined by flow cytometry. The results are expressed as means ±SD. *P < 0.05 versus the CONT group; #*P* < 0.05 versus the BSA group.

An increase in the frequency of CD4 + and CD8 + T cells as well as CD11c + IAE-IE + dendritic cells was observed in the BSA, KO, and KO + BSA groups compared with the CONT group (Figures 6A–C). However, the accumulation of all these cells, with the exception of CD8 + T cells, was higher in the KO + BSA group than in the BSA or KO groups separately. In addition, the level of Ly6G + neutrophils was increased only in the KO + BSA group (Figure 6D).

**FIGURE 7:**
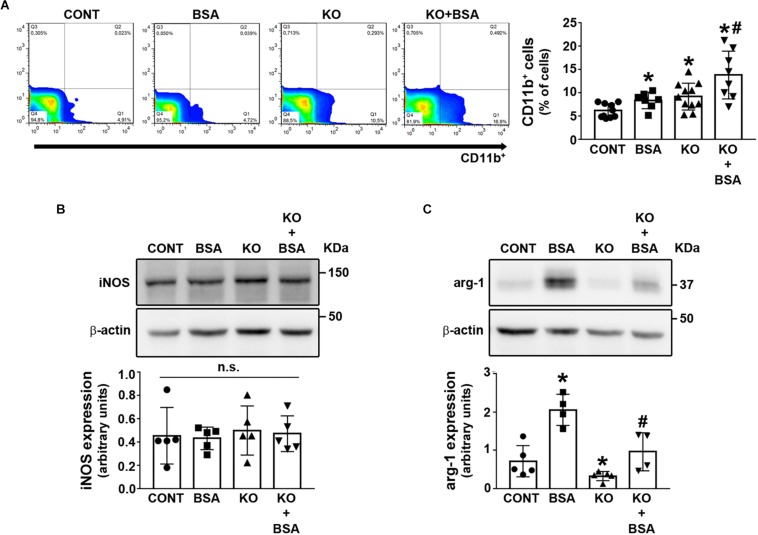
IL-4Rα deficiency abolished the increase in M2-subtype macrophages in subclinical AKI. Mice were treated as described in Figure 1. The kidneys were perfused with saline and digested with collagenase. **(A)** The level of infiltrated CD11b+ macrophages was determined by flow cytometry (n = 8). The macrophage phenotype was determined by iNOS expression [**(B)**, *n* = 5] and arginase-1 expression [**(C)**, *n* = 4] through immunoblotting of the renal cortex. iNOS, a marker of M1-subtype cells; arginase-1, a marker of M2-subtype cells. The results are expressed as means ±SD. *P < 0.05 versus the CONT group; #*P* < 0.05 versus the BSA group; n.s., not significant.

One important issue in the development of TII induced by PT albumin overload is the role of macrophages (Gorriz and Martinez-Castelao, 2012; Erkan, 2013; Portella et al., 2013; Abreu et al., 2014; Landgraf et al., 2014; Teixeira et al., 2019). Here, the CD11b + macrophage level was increased in the BSA, KO, and KO + BSA groups (Figure 7A). Further increase was observed in the KO + BSA group compared with the KO and BSA groups. To verify if the increase in total macrophages observed in the KO + BSA group correlated with possible changes in macrophage polarization, we assessed the expression of iNOS, a marker of M1-subtype macrophages, and arginase-1, a marker of M2-subtype macrophages. We observed no changes in iNOS expression in any experimental group (Figure 7B). On the other hand, arginase-1 expression was increased in the BSA group (Figure 7C). Interestingly, arginase-1 expression was higher in the KO + BSA group than in the CONT group but lower than in the BSA group, showing a decrease in polarization of macrophages to the M2 phenotype in IL4Rα^–/–^ mice.

## Discussion

Repair of tubular injury is a critical step for complete recovery from AKI (Venkatachalam et al., 2015; Strausser et al., 2018). The possible role of IL-4 in this process has been uncovered, although understanding the underlying mechanism is still a challenge. In the present study, we used a subclinical AKI animal model to study the role of IL-4 on TII induced by albumin overload. It was observed that IL-4 plays a protective role in tubular injury, and this effect is associated with PT albumin handling and regulation of the inflammatory response. These results open new avenues to understand the role of IL-4 on the course of AKI and, consequently, progression of renal disease.

In the present work, a subclinical AKI model characterized by overload of albumin in PTs without changing glomerular function was used (Ronco et al., 2012; Portella et al., 2013; Abreu et al., 2014; Landgraf et al., 2014; Teixeira et al., 2019; Vanmassenhove et al., 2019). This process results in tubular injury associated with modifications in the albumin reabsorption machinery, a pro-inflammatory response, and collagen deposition (Gorriz and Martinez-Castelao, 2012; Erkan, 2013; Portella et al., 2013; Abreu et al., 2014; Landgraf et al., 2014; Teixeira et al., 2019). Herein we observed that there is no change in apparent glomerular function measured by CCr and plasma creatinine. Ishola et al. (2006), using 129S2/Sv and C57BL/6J strains, showed that BSA overload animal model induced glomerular leakage of albumin without any changes in glomerular ultra-structure after 11 days measured by electron microscopy. The authors proposed that albumin overload in PT cells is a result of increased glomerular albumin permeability rather than by changes in glomerular ultrastructure. Nevertheless, the measurement of these parameters is not enough to assure that there is no change in glomerular function and structure in any way. In this context, it is a good animal model to study the possible effects of IL-4 on TII, independently of apparent glomerular changes. Accordingly, we observed that glomerular function was not changed in IL4Rα^–/–^ mice. However, it is important to mention that the results obtained in the present study cannot exclude possible signals generated by glomerular cells during this experimental condition. In this context, it has been proposed that injuries in glomerular structure could be transmitted to tubular segments contributing to the tubule-interstitial injury (Theilig, 2010). Although further experiments are necessary to clarify this issue.

One crucial question in the pathogenesis of TII induced by albumin overload is how PTECs sense changes in the albumin concentration in PTs. It has been proposed that megalin is a sensor for this process, triggering cellular signaling coupled to misfunction of PTECs (Caruso-Neves et al., 2006; Erkan, 2013; Peruchetti et al., 2014; Nielsen et al., 2016). Our group showed that knockdown of megalin in LLC-PK1 cells mimicked the cellular response induced by higher albumin concentration (Peruchetti et al., 2014). This process was related to apoptosis of PTECs (Caruso-Neves et al., 2006; Teixeira et al., 2019). In agreement, we have shown here that in the AKIsub model, there was a decrease in megalin expression that was associated with a pro-inflammatory phenotype and development of TII. The induction of megalin expression in this animal model reduced proteinuria and tubule damage (Cabezas et al., 2011). In agreement, Koizumi et al. (2019) showed that PT specific knockout mice for megalin were more susceptible to tubule-interstitial damage in a model of podocyte injury.

Accordingly, with the view that megalin expression is associated with the development of TII, it has been shown that megalin is involved in the genesis of different renal diseases (Piwon et al., 2000; Christensen et al., 2003; Batuman, 2007; Nakhoul and Batuman, 2011; Vicinanza et al., 2011; Gaide Chevronnay et al., 2014; Eshbach and Weisz, 2017; Wang et al., 2017; Cez et al., 2018; Larsen et al., 2018; Peruchetti et al., 2018; Silva-Aguiar et al., 2018). Larsen et al. (2018) showed that megalin is the antigen target of human kidney anti-brush border antibody that causes a primary renal tubulointerstitial disease (ABBA disease). Furthermore, megalin has been proposed to be involved in Fanconi’s syndrome and Dent’s syndrome, correlated with the increase in the pro-inflammatory response and TII (Piwon et al., 2000; Christensen et al., 2003; Batuman, 2007; Nakhoul and Batuman, 2011; Vicinanza et al., 2011; Gaide Chevronnay et al., 2014; Eshbach and Weisz, 2017; Wang et al., 2017; Cez et al., 2018).

But the mechanism involved in the decrease of megalin expression is not clear yet. An important link could be TGF-β secretion. Diwakar et al. (2007) using OK cells showed that albumin induces TGF-β secretion. Recently, Cabezas et al. (2019) using LLC-PK1 cells showed that TGF-β decreases megalin expression. Accordingly, we observed an increase in the TGF-β level in the BSA groups. On the other hand, the decrease in megalin expression was only observed when TII was induced despite an increase in the TGF-β level in IL4Rα^–/–^ mice. In addition, IL-4 modulates the albumin uptake in LLC-PK1 cells only when the cells are exposed to a higher albumin concentration. These results indicate megalin-mediated albumin endocytosis is a component of a complex mechanism involved in the pathogenesis of TII induced by albumin overload.

Therefore, the modulation of albumin endocytosis by IL-4 is an attractive hypothesis in understanding the mechanism underlying the modulation of TII induced by albumin overload. Only few studies have shown the possible correlation between epithelial cell protein endocytosis and IL-4. Van Den Berg et al. (2002) showed that IL-4 increases intracellular trafficking of proteins in glomerular visceral epithelial cells. Olsan et al. (2015), using human autosomal-dominant polycystic kidney disease tissues, showed that STAT6 pathway activation induces an increase in renal pIgR, which is involved in renal cell IgA transport. Our data indicate that IL-4 has a protective effect against the decrease in megalin expression and PT albumin endocytosis induced by higher albumin concentration. This mechanism involves activation of the JAK3/STAT6 pathway and seems to be involved in a protective role of IL-4 on TII.

How PTEC mediates TII induced by albumin overload is not clear. Some studies have shown that PTECs could secrete cytokines and chemokines, which could mediate activation of the pro-inflammatory response (Zoja et al., 1998; Donadelli et al., 2003; Takaya et al., 2003; Tang et al., 2003; Reich et al., 2005; Pearson et al., 2008; Gorriz and Martinez-Castelao, 2012; Erkan, 2013; Portella et al., 2013; Landgraf et al., 2014; Teixeira et al., 2019). These observations bring a new concept to PTECs: they could be a sensor, transductor, effector, and the final target of different cytokines and chemokines involved in the development of TII. We showed that IL-4 binds to the receptor, triggers a cellular response, and modulates PT endocytosis during the development of TII. We propose that besides immune cells, PTECs could work as a transducer in the effect of IL-4 on albumin endocytosis, pro-inflammatory response, and the development of TII. Accordantly, Breda et al. (2019) proposed that PTECs could work as APCs, inducing an inflammatory phenotype in CD4 + T cells.

Data from the literature indicate that macrophage infiltration during the pathogenesis of AKI is a critical process in the development and repair of TII (Huen and Cantley, 2017; Lee et al., 2017). M1 macrophage phenotypes are predominant at the early phase and switch to M2 phenotypes at a later phase of AKI (Huen and Cantley, 2017; Lee et al., 2017). Using a subclinical AKI model, our group showed that tubular injury induced by albumin overload encompasses a pro-inflammatory response followed by polarization of the M2 macrophage phenotype, an increase in renal TGF-β level, and collagen deposition (Landgraf et al., 2014; Teixeira et al., 2019). Here, we observed an increase in total macrophage infiltration but a decrease in M2 phenotypes associated with a decrease in collagen deposition in TII-induced IL4Ra^–/–^ mice. In agreement, it has been proposed that IL-4 participates in the resolution phase, leading the polarization of M2 phenotype macrophages and promoting the recovery from AKI (Cao et al., 2011; Yan et al., 2015; Liang et al., 2017; Zhang et al., 2017). Furthermore, Lee et al. (2011) showed that macrophage ablation after established AKI worsened the tubular injury due to reduced M2 phenotype.

In parallel with the decrease in M2 macrophages, an increase in IL-6 and IL-17 cytokines was observed, as well as infiltrated CD4 + T cells, neutrophils, and dendritic cells in subclinical AKI developed in IL4Rα^–/–^ mice. Interestingly, a further increase in TGF-β level was observed in IL4Rα^–/–^ mice. Chung et al. (2018) using macrophage TGF-βRII^–/–^ mice showed that TGF-β works as a chemoattractant mediating renal fibrosis and, consequently, the progression of renal disease. On the other hand, we showed that despite the increase in TGF-β level, there was a decrease in M2 phenotype macrophages associated with a reduction in collagen deposition and worsening TII. These results indicate that the resolution phase involves a more complex network than has been postulated. Probably, the final effect of the TGF-β level involves several coordinated actions. In agreement, it has been shown that IL-4 induces renal fibrosis depending on polarization to M2 phenotype macrophages and the activation of marrow-derived fibroblasts (Liang et al., 2017; Zhang et al., 2017). Further experiments are necessary to clarify this issue.

Our results indicate that IL-4 plays an important role in the balance between the initial and resolution phases of the inflammatory response. In a subclinical AKI model developed in IL4Rα^–/–^ mice, there is an increase in the Th1 response associated with preponderance in M1 phenotype macrophages over the M2 phenotype, extending the early phase and delaying the resolution phase. Although, these conclusions are limited since we used one time point (day 7 of subclinical AKI) what does not allow us to determine longitudinal events of initial and resolution phases.

**FIGURE 8:**
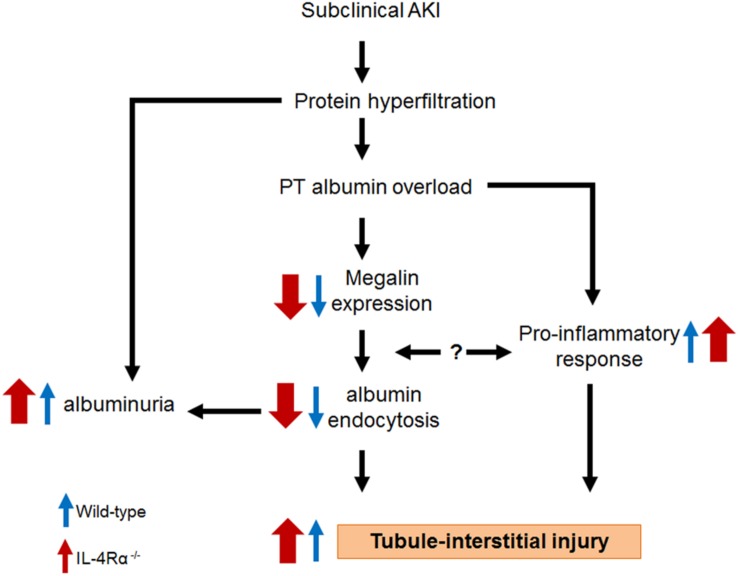
Proposed model for the role of IL-4 on the tubule-interstitial injury induced by albumin overload in the proximal tubule. Blue arrow, wild-type mice. Red arrows, IL4Rα^–/–^ mice.

Based on the results obtained in the present work together with those published previously by other authors, we postulate a mechanism underlying the effect of IL-4 on the TII induced by albumin overload (Figure 8). IL-4 plays an important role in development of TII due to its action: (1) on the megalin expression, albumin endocytosis and, consequently on the albuminuria. This mechanism involves activation of JAK3/STAT6; (2) in modulating pro-inflammatory response. In this case, IL-4 attenuates the increase in cortical pro-inflammatory cytokines IL-6, IL-17 as well as the increase in TGF-β, a pro-fibrotic cytokine. Other possibility could be the direct effect of pro-inflammatory cytokines on the PT megalin expression and, consequently, on the albumin endocytosis. This process could form a dangerous loop aggravating TII. In conclusion, IL-4 attenuates the effect of albumin overload in both megalin expression and pro-inflammatory response what could explain why IL4Rα^–/–^ mice has a more severe TII.

## Data Availability Statement

All datasets generated for this study are included in the article/supplementary material.

## Ethics Statement

The animal study was reviewed and approved by the Institutional Ethics Committee of the Federal University of Rio de Janeiro (protocol number IBCCF098).

## Author Contributions

PA and CC-N conceived and designed the study. DP, JS-F, RS-A, DT, MS, and MH contributed to acquisition of the data. DP, JS-F, RS-A, DT, CT, AP, and CC-N contributed to analysis and interpretation of the data. D-PB and CC-N wrote the first draft of the manuscript. All authors contributed to revision of the manuscript, read, and approved the submitted version.

## Conflict of Interest

The authors declare that the research was conducted in the absence of any commercial or financial relationships that could be construed as a potential conflict of interest.

## Footnotes

1 www.graphpad.com
